# Endothelial glycocalyx in different flow regions of the trabecular outflow pathway in bovine eyes

**DOI:** 10.3389/fcell.2025.1569569

**Published:** 2025-04-25

**Authors:** Hoi-Lam Li, Neil Liu, Shayna Sosnowik, Michelle Yung, Haiyan Gong

**Affiliations:** ^1^ Ophthalmology, Boston University School of Medicine, Boston, MA, United States; ^2^ Department of Ophthalmology, MedVet, Columbus, Worthington, OH, United States

**Keywords:** endothelial glycocalyx, trabecular outflow pathway, aqueous plexus, giant vacuoles, I-pores, B-pores, collector channel, episcleral vein

## Abstract

**Background:**

Glycocalyx is a hair-like structure covering the endothelium of the aqueous outflow pathway. While trabecular outflow is segmental circumferentially around the eye, regional differences in glycocalyx morphology remain largely unexplored. This study investigated glycocalyx variations in the different structures along the trabecular outflow pathway in high-flow (HF) and low-flow (LF) regions of bovine eyes.

**Methods:**

Enucleated bovine eyes (n = 8) were perfused with fluorescein to identify HF and LF regions. The glycocalyx was labeled with Alcian Blue 8GX, and radial wedges from the anterior chamber angles of both HF and LF regions were processed for transmission electron microscopy. Glycocalyx thickness and coverage were quantified using ImageJ and compared between different outflow pathway locations in HF and LF regions. Glycocalyx measurements at intracellular (I-pores) and border pores (B-pores), the percentage of glycocalyx-unfilled pores, as well as the percentage of giant vacuoles (GVs) with and without I-pores with glycocalyx lining the inner membrane were evaluated.

**Results:**

Glycocalyx thickness and coverage did not differ significantly between HF and LF regions. However, thickness progressively increased from the proximal (trabecular meshwork) to the distal (episcleral veins) outflow pathway. In both I-pores and B-pores, the glycocalyx was present near the basal opening, edge, and center of the pores, with thickness increasing toward the center. No significant differences in the percentage of glycocalyx-filled pores were observed between HF and LF regions. However, the percentage of GVs with I-pores exhibiting glycocalyx lining the inner cellular membrane was significantly higher (100%) than that of those without I-pores (16%).

**Conclusion:**

No regional differences were found between HF and LF regions, but glycocalyx thickness progressively increased from the proximal to the distal outflow pathway, potentially reflecting varying shear stress conditions. The significantly higher percentage of GVs with I-pores containing glycocalyx lining the inner cellular membrane compared to those without I-pores suggests a relationship between aqueous outflow dynamics and glycocalyx synthesis. These findings provide a morphological basis for future research on glycocalyx alterations in glaucoma and their impact on outflow resistance.

## 1 Introduction

The glycocalyx is a dense, bush-like layer of proteoglycans and glycoproteins, interwoven with soluble components, such as albumin and hyaluronan. It coats the surface of endothelial or epithelial cells (Weinbaum et al., 2007; Reitsma et al., 2007). Glycocalyx has been well-documented on vascular endothelial surfaces (van den Berg et al., 2003; Haldenby et al., 1994; Chevalier et al., 2022; Yen et al., 2012), where it acts as a mechanosensor, detecting fluid shear stress to mediate vascular protection (Weinbaum et al., 2007; Reitsma et al., 2007). Additionally, the glycocalyx acts as a permeability barrier, restricting the movement of large molecules out of the bloodstream (Kutuzov et al., 2018; van Haaren et al., 2003). Our previous studies identified the presence of glycocalyx along the endothelial cells of the trabecular (conventional) outflow pathway in bovine, monkey, and human eyes, resembling that of vascular endothelium and suggesting potentially similar functions (Yang et al., 2014; Sosnowik et al., 2022a). On the other hand, while laser damage to the proximal structure (trabecular meshwork) inhibits aqueous humor drainage, the entire outflow pathway exhibited minimal to absent glycocalyx (Sosnowik et al., 2022a). These findings suggest a relationship between outflow dynamics and the synthesis of the glycocalyx. While glycocalyx structure and function have been extensively characterized in the vasculature, its morphology and role in the aqueous outflow pathway have not been extensively investigated. Therefore, this study focused on the morphology of glycocalyx along the trabecular outflow pathway.

The trabecular outflow pathway serves as the main route for aqueous humor drainage from the eye (Bill, 1966; Bill and Phillips, 1971). Aqueous humor flows through the proximal outflow pathway, which consists of the trabecular meshwork (TM) and Schlemm’s canal (SC), and continues through the distal outflow pathway, including the collector channels (CCs), the intrascleral veins and episcleral veins (ESVs) in human eyes (Freddo et al., 2022; Tripathi, 1977). Similar structures have been identified in bovine eyes, where SC is replaced by the aqueous plexus (AP), a functional analog (Tripathi, 1971a). Tight junctions connect the endothelial cells of SC/AP, preventing easy egress of aqueous humor from the TM into SC/AP (Bhatt et al., 1995; Raviola and Raviola, 1981).

There are two routes to enter SC/AP. In the first route, a pressure gradient from the TM to SC causes the formation of giant vacuoles (GVs) along the endothelium of SC/AP. As GVs enlarge, their lining cell membranes become thinner, and transcellular or intracellular pores (I-pores) form, allowing aqueous humor to flow (Tripathi, 1971b; Swain et al., 2022). In the second route, aqueous humor may enter SC/AP through the openings between two adjacent endothelial cells, called paracellular or border pores (B-pores) (Lai et al., 2019; Swain et al., 2021). Glycocalyx is also observed along the I-pores and B-pores in bovine, monkey, or human eyes (Yang et al., 2014; Sosnowik et al., 2022a). Depending on the thickness (or length), and distribution of the glycocalyx along the pores, the glycocalyx may either fully occupy these pores (“filled pores”) or leave them opening (“unfilled pores”) (Yang et al., 2014; Sosnowik et al., 2022a). The glycocalyx has been hypothesized to modulate resistance in microvessels up to 30 microns in diameter (Yang et al., 2014; Weinbaum et al., 2003). A glycocalyx-filled pore is likely to have significantly higher flow resistance than an empty pore. Since trabecular outflow is uneven or segmental across human eyes (Huang et al., 2017; Cha et al., 2016; Chang et al., 2014; Vranka et al., 2015), with high-flow (HF) and low-flow (LF) regions, glycocalyx-filled pores may play a role in regulating aqueous outflow resistance (Yang et al., 2014). However, the percentage of filled or unfilled pores in different flow regions and their role in regulating segmental flow remains unknown.

Segmental trabecular outflow has also been observed in bovine eyes (Huang et al., 2016), but differences in glycocalyx properties and distribution between HF and LF regions, as well as their relationships with pore occupancy and regional flow variations remain unexplored. To address the knowledge gaps, this study aims to investigate glycocalyx distribution and thickness variations across different structures of the trabecular outflow pathway in HF and LF regions of bovine eyes.

## 2 Materials and methods

### 2.1 Ocular perfusion

**FIGURE 1:**
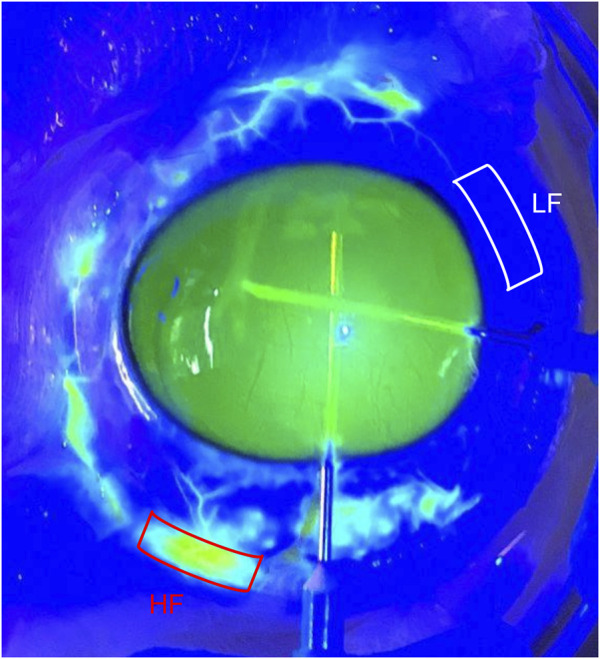
Segmental flow in bovine eyes following fluorescein perfusion. The white box indicates the areas without fluorescent episcleral veins (ESVs), a low-flow area, and the red box indicates fluorescent ESVs, a high-flow area.

Fresh enucleated bovine eyes (n = 8) were obtained from a local abattoir and delivered on ice within 6 h post-mortem. All the eyes were perfused, labeled, fixed, and embedded following a previously described protocol (Sosnowik et al., 2022a; Sosnowik et al., 2022b). Briefly, after establishing baseline outflow facility at 15 mmHg for 30 min, 0.1% fluorescein in Dulbecco’s phosphate-buffered saline (Life Technologies, Grand Island, NY, United States) containing 5.5 mM of D-glucose (DPBS) was exchanged (5 mL) and perfused into the anterior chamber for 10 min at 15 mmHg. The distribution of fluorescein in the ESVs was imaged under illumination with cobalt blue light to identify the HF and LF regions (Figure 1). To fix the tissue and stain the glycocalyx, all eyes were exchanged and perfusion-fixed for 1 h at 15 mmHg with modified Karnovsky’s fixative (1% glutaraldehyde and 4% paraformaldehyde in DPBS, containing 30 mmol/L MgCl_2_) with 0.05% (*w/v*) Alcian Blue 8GX, then immersion-fixed with the same fixative overnight.

### 2.2 Sample preparation

**FIGURE 2:**
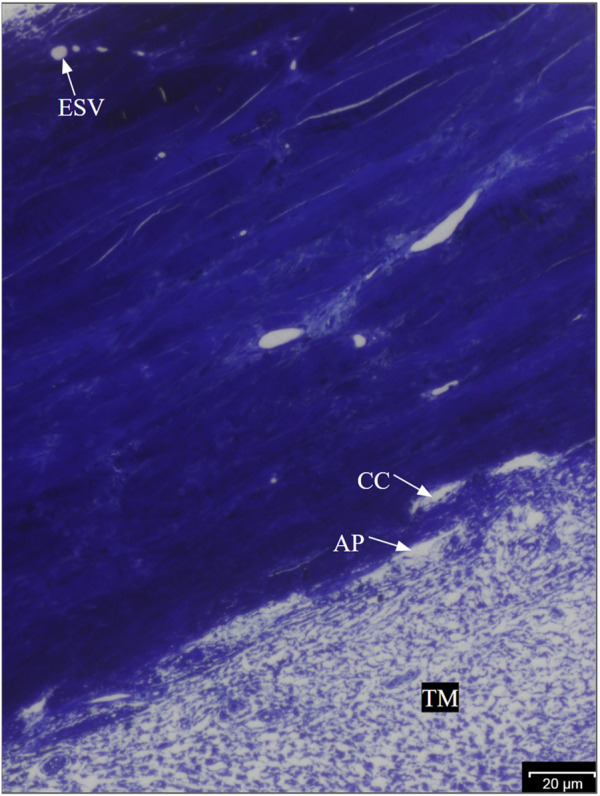
Anatomy of the bovine trabecular outflow pathway. Aqueous humor flows sequentially through the TM, trabecular meshwork; AP, aqueous plexus; CCs, collector channels; and ESVs, episcleral veins in bovine eyes.

Regions showing fluorescence in ESVs were identified as HF regions, and those without fluorescence were identified as LF regions (Figure 1) and dissected radially. All wedges were post-fixed with 1% osmium tetroxide and 1% lanthanum III nitrate hydrate solution for 2 h. The samples were then stained with 1.5% uranyl acetate for 1 h. The wedges were dehydrated sequentially in ethanol with increasing concentrations (50%, 70%, 80%, 95%, and 100%). After washing with propylene oxide, the wedges were infiltrated with a 1:1 propylene oxide and Epon-Araldite mixture for 3 h and then infiltrated with 100% Epon-Araldite overnight. Finally, the samples were embedded in 100% Epon-Araldite. Semi-thin (4 μm) sections were cut and stained with 1% toluidine blue. The outflow pathway structures were identified under ×20 magnification using light microscopy (Figure 2), and ultra-thin (70 nm) sections were cut using a microtome.

### 2.3 Electron microscopy and data analysis

At least 10 images of each structure: trabecular meshwork beams (TMBs), AP, CCs, and ESVs from both HF and LF regions of each eye were taken using transmission electron microscopy (JEOL JEM-1400 Flash). Each structure included a minimum of five images at ×8,000 and ×20,000 magnifications to measure glycocalyx coverage and thickness, respectively. Glycocalyx coverage was defined as the percentage of the endothelial surface covered by glycocalyx relative to the total length of the endothelial surface imaged. Glycocalyx thickness was measured as the perpendicular distance from the base of the glycocalyx bundle to the end of its tallest strand, as previously described (Sosnowik et al., 2022a). Both glycocalyx coverage and thickness were measured twice by two independent investigators using ImageJ software, with less than 10% variability. The average of these measurements was used for further analysis.

**FIGURE 3:**
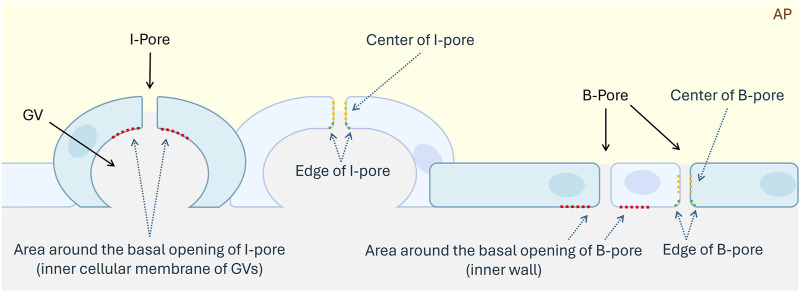
Schematic diagram of I-pores and B-pores in the inner wall of the aqueous plexus. Blue cells represent the inner wall cells of the AP, aqueous plexus. Red dotted lines indicate the region around the basal openings of I-pore and B-pore. Green dotted lines indicate the edges of pores. Yellow dotted lines represent the centers of pores. GV, Giant Vacuole.

GVs, I-pores, and B-pores in both HF and LF regions were identified by observing continuous sections using transmission electron microscopy. I-pores were generally observed with GVs, while B-pores were located between two adjacent endothelial cells. The percentage of GVs with glycocalyx lining the inner membrane relative to the total number of GVs was calculated for each eye in both flow regions. The percentage of glycocalyx-filled GVs was compared between GVs with and without I-pores. For both I-pores and B-pores, glycocalyx thickness was measured at three locations: the area around the basal opening, at the edge, and at the center of the pores (Figure 3). The percentage of unfilled I-pores and B-pores was analyzed.

### 2.4 Statistical analysis

All data are presented as mean ± SEM. Statistical analysis was performed using GraphPad Prism (version 9.5.1, GraphPad Software, San Diego, CA). A two-way ANOVA followed by Tukey’s multiple comparisons test was used to compare the glycocalyx coverage and thickness at HF and LF regions and different outflow pathway structures. Two-tailed paired t-tests were used for comparisons between the percentage of glycocalyx lining the inner membrane in GVs with and without I-pores. Fisher’s exact test was used to analyze associations between pore types, glycocalyx filling, and flow regions. A *p*-value of <0.05 was considered statistically significant (*p < 0.05; **p < 0.01; ***p < 0.001; ****p < 0.0001). A normality test, specifically the Shapiro-Wilk test, was performed on all data prior to analysis. This ensured that all data used in the two-way ANOVA and t-test passed the normality test (*p* > 0.05). In contrast, all data used in Fisher’s exact test failed the normality test (*p* < 0.05).

## 3 Results

### 3.1 Glycocalyx morphology and distribution along the trabecular outflow pathway

**FIGURE 4:**
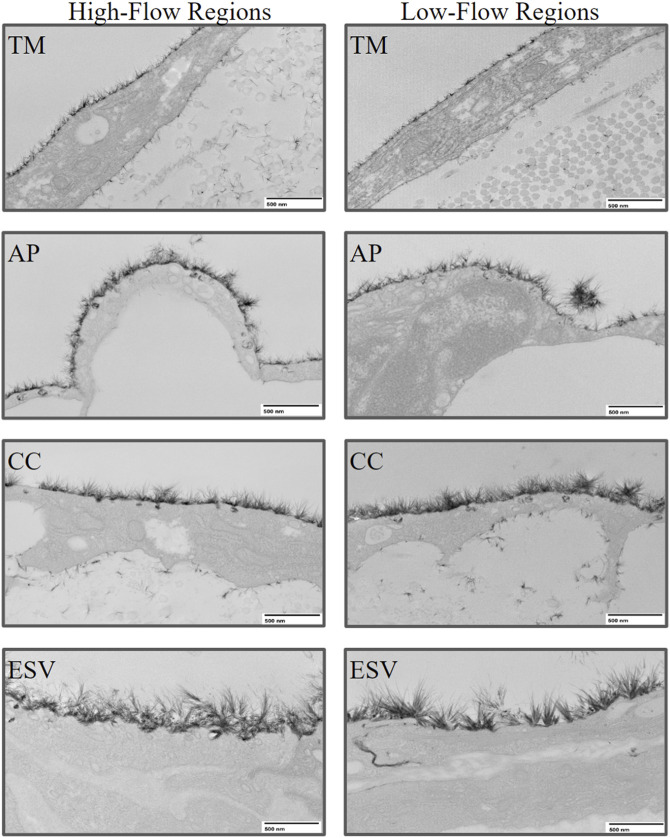
Representative images of glycocalyx lining the endothelium of trabecular outflow pathway structures in high-flow and low-flow regions. The glycocalyx was imaged at 8,000x magnification using transmission electron microscopy. TM, Trabecular Meshwork; AP, Aqueous Plexus; CC, Collector Channel; ESV, Episcleral Vein.

**FIGURE 5:**
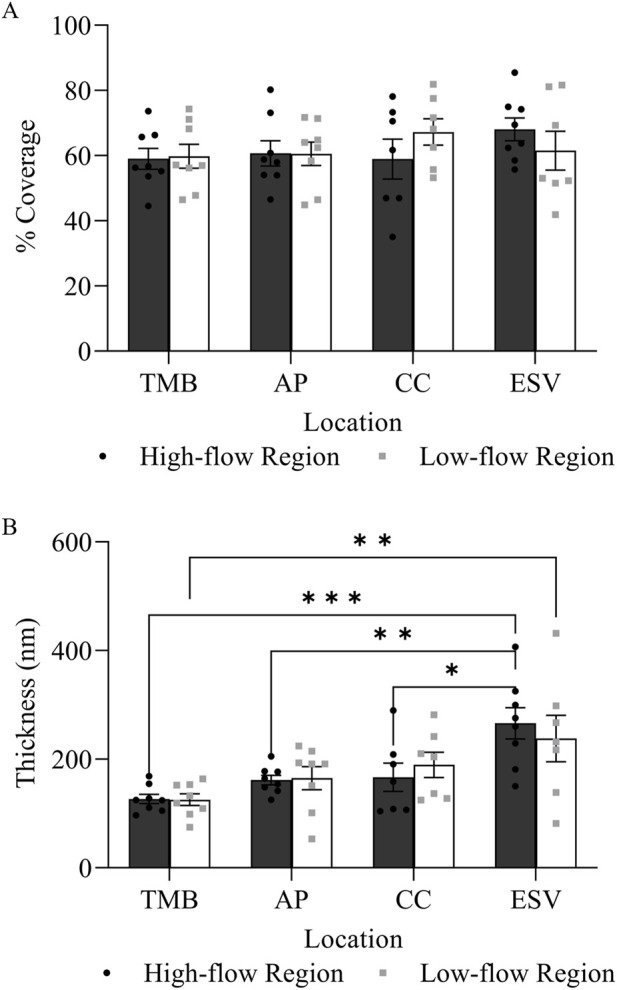
Glycocalyx distribution and thickness in high-flow and low-flow regions along the trabecular outflow pathway. **(A)** There was no significant difference in the percentage coverage of glycocalyx between flow regions at each outflow pathway location. **(B)** There was no significant difference in glycocalyx thickness between the high-flow and low-flow regions at each outflow pathway location. However, there was a significant progressive increase in glycocalyx thickness from the proximal (TMB) to distal (ESV) outflow pathway in both high-flow and low-flow regions.

Glycocalyx was observed along the endothelium of different structures along the trabecular outflow pathway, including the TM, AP, CCs, and ESVs (Figure 4). No significant difference was found in the percentage coverage of glycocalyx between HF and LF regions or across different structures (*p* > 0.05, Figure 5A). No significant differences in glycocalyx thickness were observed between HF and LF regions for each outflow pathway structure individually (*p* > 0.05, Figure 5B). However, a significant progressive increase in glycocalyx thickness was noted from the proximal (TMB) to the distal (ESV) outflow pathway in both HF and LF regions. The overall increase in average glycocalyx thickness from the TM to ESVs was 139.30 ± 26.18 nm (49% ± 6%, *p* < 0.001) in the HF region and 110.35 ± 45.61 nm (33% ± 14%, *p* < 0.01) in the LF region.

### 3.2 Glycocalyx morphology and distribution in GVs and I-pores

**FIGURE 6:**
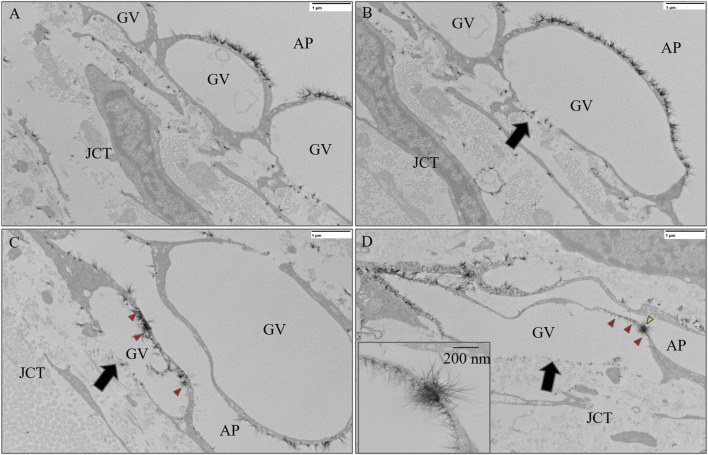
Representative images of giant vacuoles (GVs) with and without glycocalyx lining the inner cellular membrane. **(A)** A GV without a basal opening or I-pore and **(B)** a GV with a basal opening (black arrow) but no I-pore and no glycocalyx lining the inner cellular membrane. **(C)** A GV with a basal opening (black arrow) but without an I-pore with glycocalyx (red arrowheads) lining the inner cellular membrane. **(D)** A GV with an I-pore (green arrowhead) and a basal opening (black arrow), as well as with glycocalyx lining the inner cell membrane (red arrowheads). GV, Giant Vacuole; AP, Aqueous Plexus; JCT, Juxtacanalicular Tissue.

**FIGURE 7:**
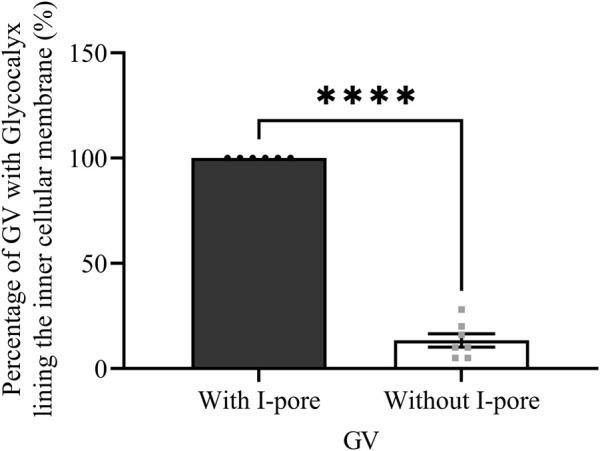
The percentage of GVs with and without I-pores with glycocalyx lining the inner cellular membrane. A significantly higher percentage of GVs (100%) with I-pores displayed glycocalyx lining the inner cellular membrane compared to GVs without I-pores (16%).

A total of 235 GVs were examined across six eyes, including 12 and 10 GVs with I-pores in HF and LF regions, respectively. In HF regions, 53% of I-pores were unfilled, and in LF regions, 60% were unfilled, with no significant association found between pore filling and flow region (*p* > 0.05). Among the GVs, some did not exhibit glycocalyx lining the inner cellular membrane (Figures 6A, B), while others did (Figures 6C, D). Our results showed that all GVs with I-pores had glycocalyx lining the inner membrane (100%), significantly higher than GVs without I-pores by 86% ± 4% (*p* < 0.0001, Figure 7).

**FIGURE 8:**
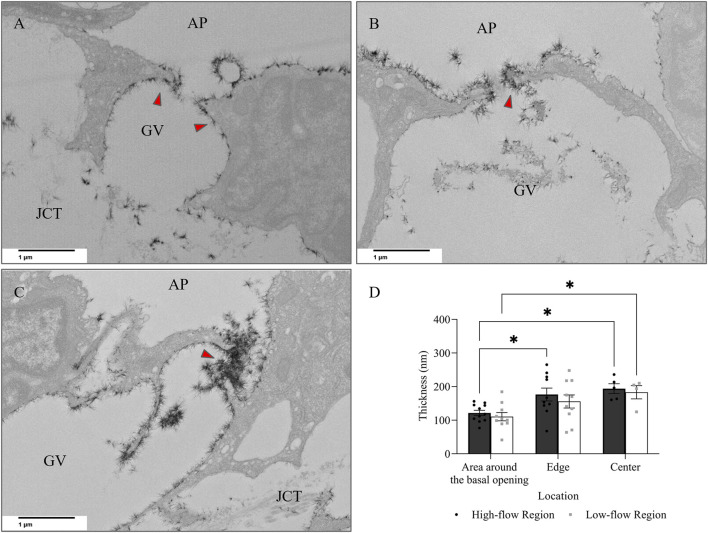
Glycocalyx thickness along I-pores. Representative images of glycocalyx (red arrow) lining: **(A)** around the basal opening, **(B)** along the edge, and **(C)** in the center of I-pores **(D)**. No significant difference in glycocalyx thickness associated with I-pores was observed between flow regions. However, glycocalyx thickness increased from the area around the basal opening to the center of the I-pores in both flow regions. GV, Giant Vacuoles; AP, Aqueous Plexus; JCT, Juxtacanalicular Tissue.

When comparing glycocalyx thickness at the area around the basal opening (Figure 8A), the edge (Figure 8B), and the center (Figure 8C) of I-pores, no significant differences were observed between the HF and LF regions. However, glycocalyx thickness increased significantly from the area around the basal opening to the center of I-pores by 72.32 ± 14.56 nm (59% ± 12%, *p* < 0.05) in the HF region and 72.77 ± 19.77 nm (66% ± 18%, *p* < 0.05) in the LF region (Figure 8D).

### 3.3 Glycocalyx morphology and distribution in B-pores

**FIGURE 9:**
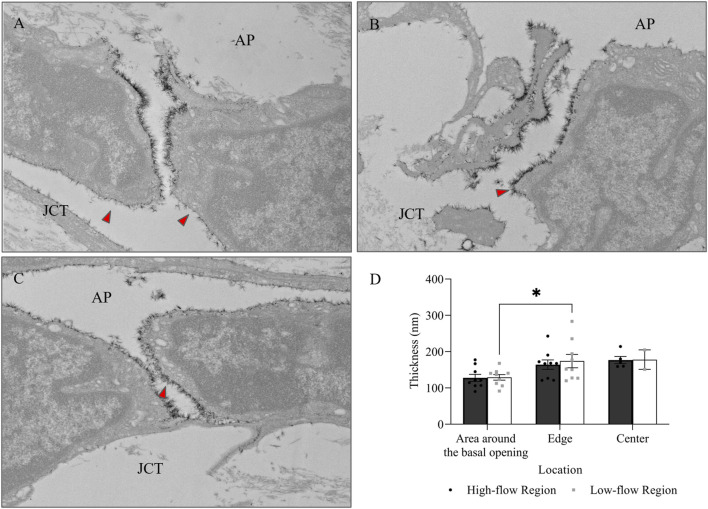
Glycocalyx thickness along B-pores. Representative images of glycocalyx (red arrow) lining: **(A)** around the basal opening, **(B)** along the edge, and **(C)** in the center of B-pores. **(D)** No significant difference in glycocalyx thickness was found between flow regions. However, the thickness increased from the area around the basal opening to the edge of the B-pores in the low-flow region. AP, Aqueous Plexus; JCT, Juxtacanalicular Tissue.

In addition to I-pores, 9 and 11 B-pores were identified in HF and LF regions, respectively, across six eyes. The percentage of unfilled B-pores was 67% in the HF region and 60% in the LF region, with no significant association found between the flow region and pore filling (*p* > 0.05). For all 20 B-pores, glycocalyx thickness showed an increasing trend from the area around the basal opening (Figure 9A) to the edge (Figure 9B) and center (Figure 9C) of the B-pores. The thickness at the edge was significantly higher than at the area around the basal opening in the LF region by 44.88 ± 18.48 nm (35% ± 14%, *p* < 0.05, Figure 9D). However, no significant differences were found between the HF and LF regions at any location.

**FIGURE 10:**
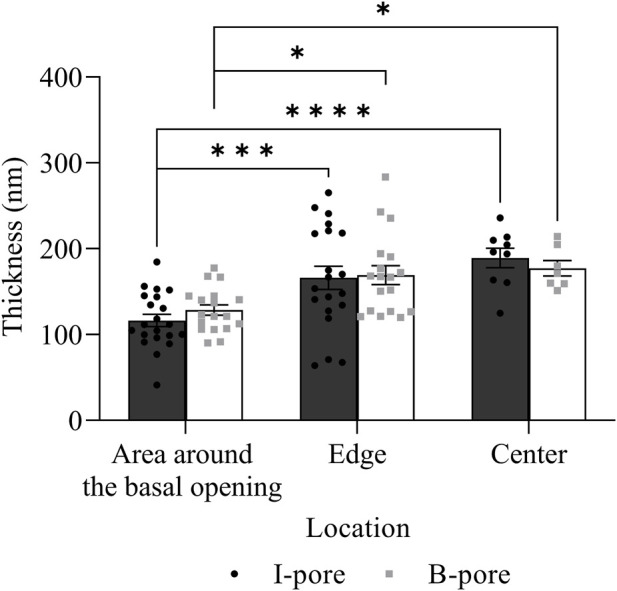
Glycocalyx thickness along both I- and B-pores. The glycocalyx thickness increased from the area around the basal opening to the center of both I- and B-pores. However, no significant differences were observed between the two types of pores at any locations.

No significant differences in glycocalyx thickness were found between HF and LF regions in either I-pores or B-pores, so data from both flow regions were combined for comparison (Figure 10). The glycocalyx thickness significantly increased from the area around the basal opening to the center of pores in I-pores by 72.88 ± 11.26 nm (63% ± 10%, *p* < 0.0001) and B-pores by 48.68 ± 9.12 nm (38% ± 7%, *p* < 0.05). No significant difference in glycocalyx thickness was found between I-pores and B-pores at any individual location (*p* > 0.05).

## 4 Discussion

This study investigated glycocalyx variations in the different structures along the trabecular outflow pathway in HF and LF regions of bovine eyes. We confirm that glycocalyx thickness progressively increased from proximal to distal regions of the trabecular outflow pathway in bovine eyes, consistent with previous observations in monkey and human eyes (Sosnowik et al., 2022a). The organization and structure of glycocalyx are influenced by shear forces. In particular, the expression of hyaluronan, a key structural component of the glycocalyx, increases with elevated laminar shear stress (Wang et al., 2020), reflecting the glycocalyx’s role in sensing mechanical forces. Regions subjected to higher shear stress are known to exhibit thicker glycocalyx layers (Arisaka et al., 1995), likely corresponding to higher fluid flow rates. In the TM, aqueous humor flows through the intertrabecular spaces between TMBs before passing through pores in the inner wall of the AP, disrupting the laminar flow and reducing shear stress. Additionally, compared to CCs and ESVs, the TM and AP have a larger open space or diameter and lower resistance (Johnson, 2006; Gong and Francis, 2014). Hence, aqueous humor may experience less turbulence and smoother laminar flow in TM and AP, potentially resulting in a thinner glycocalyx layer in TM and AP compared to CC and ESVs.

However, for glycocalyx coverage, our results showed comparable coverage in TM, AP, CC, and ESV, consistent with the findings in normal monkeys. Remarkably, damage to the TM induced a significant decrease in glycocalyx coverage from TM to ESV (Sosnowik et al., 2022a). Other studies indicate that glycocalyx coverage diminishes in aged or diseased resected vessels (Targosz-Korecka et al., 2017; Salmon et al., 2012) and in cultured vascular endothelial cells that mimicked aged or unhealthy arteries (Mahmoud et al., 2021). Since glycocalyx coverage remains stable under healthy or normal conditions, this suggests that healthy endothelial cells produce a consistent glycocalyx coverage, which may serve a protective role in maintaining endothelial health.

To further explore the relationship between glycocalyx morphology and aqueous outflow through high-resistance, endothelium-lined structures, we examined glycocalyx morphology and distribution in the inner wall of the AP, specifically, in GVs and pores in both HF and LF regions. GVs form when inner wall endothelial cells deform under pressure (Gong et al., 1996), with I-pores often developing in regions where the cellular lining becomes thinner (Swain et al., 2021). Given that fluid flow through these structures induces shear stress, this may stimulate glycocalyx formation (Wang et al., 2020; Arisaka et al., 1995). Our findings revealed that 100% of GVs with I-pores exhibited glycocalyx lining on the inner cellular membrane, consistent with the observation in monkey eyes (Sosnowik et al., 2022a). This supports the idea that glycocalyx formation is associated with aqueous humor flow. However, we also identified glycocalyx lining on the inner cellular membrane in 16% of GVs without I-pores. This could be due to two possibilities: the first is our experimental limitations, as the morphology of the entire GVs could not be fully captured without evaluating tissue through serial sections. It is possible that pores were present in the unexamined adjacent sections, which contained portions of these GVs. The second possibility is because fixed samples were used in this study, we can only observe the structure at the moment of fixation, not time-dependent changes. It is possible that some GVs initially possessed pores that later closed at the time of fixation, while flow-induced glycocalyx persisted. Further studies using serial block-face scanning electron microscopy (SBF-SEM) and live-cell imaging may help address these limitations.

The inner wall of the AP is comprised of a continuous layer of endothelial cells connected by tight junctions, restricting aqueous passage (Gong et al., 1996). The aqueous humor must, therefore, flow from the TM into the AP via I-pores or B-pores (Lai et al., 2019; Ethier et al., 1998; Braakman et al., 2015). Our results showed an increase in glycocalyx thickness from the areas around the basal opening to the center of I-pores. B-pores also exhibited a trend of increasing glycocalyx thickness from the basal cell membrane to the center of pores. These findings are likely due to the increasing shear stress with the decrease in the diameter of the region through which aqueous humor flows from GV to I-pore and from the JCT to B-pore. As previously noted, shear stress has been documented to stimulate glycocalyx development (Zeng and Tarbell, 2014). Over time, continued glycocalyx growth could lead to pore filling, potentially increasing resistance and further impeding aqueous humor flow, possibly contributing to segmental differences in outflow. However, we found no significant association between the percentage of unfilled pores and flow regions. This finding may be due to our small sample size or interindividual variability in glycocalyx expression. Additionally, it is possible that the distribution of filled or unfilled pores is influenced by transient hemodynamic changes that were not captured at the time of fixation. Further investigations with larger sample sizes and serial-sectioned images may provide a more comprehensive understanding of the role of filled or unfilled pore distribution and its relationship to different flows.

We observed no significant differences in glycocalyx thickness between the LF and HF regions 1) at the I-pores, 2) at the B-pores, or 3) along the outflow pathway structures. This contradicts our initial thoughts that HF regions would exhibit increased aqueous outflow, greater shear forces, and correspondingly thicker glycocalyx layers. While the thickness of the glycocalyx associated with pores does not appear to contribute to the segmental nature of aqueous outflow, previous studies have reported a higher number of pores in HF regions than in LF regions (Swain et al., 2022; Braakman et al., 2015). This theoretically allows greater aqueous humor volume to enter the AP in HF regions. Therefore, the higher fluorescence intensity in HF regions may result from a greater number of pores rather than a higher flow rate through individual pores. Additionally, several possible explanations may account for this unexpected finding. First, glycocalyx turnover is highly dynamic. Gomez Toledo et al. summarized the mechanisms of endothelial glycocalyx turnover, which include synthesis, intracellular degradation, surface proteolytic shedding, and glycan shedding. They suggested that glycocalyx components are regulated by both synthesis and degradation, interacting with various environmental changes (Gomez et al., 2025). Therefore, transient variations in flow may not necessarily lead to sustained differences in glycocalyx thickness at a single time point (Tripathi, 1971a). Second, research has shown that different surface components contribute to varying portions of the total thickness of the glycocalyx and diffusion coefficient (Gao and Lipowsky, 2010). Therefore, other compensatory mechanisms, such as endothelial cell surface modifications or localized shear stress adaptation, may regulate outflow resistance independently of glycocalyx thickness (Zeng and Tarbell, 2014). These mechanisms may influence glycocalyx formation and turnover by affecting the shear stress environment, which could alter the rates of synthesis and degradation. Additionally, it is possible that glycocalyx composition or its correlation with other molecules, rather than thickness alone, plays a more critical role in modulating segmental outflow (Weinbaum et al., 2003). For example, various correlations have been reported between the glycocalyx and different molecules such as matrix metalloproteinases (Cooper et al., 2018), fibroblast growth factors (Yang et al., 2017), and nitric oxide (Yen et al., 2015; Pahakis et al., 2007; Florian et al., 2003). Future studies employing advanced imaging techniques and molecular analyses may help clarify these relationships.

Glycocalyx thickness remained comparable between the HF and LF regions, not only in the pores, but also from the TM through the ESVs, implying similar shear stress levels. One possible explanation is that glycocalyx homeostasis is maintained through regulatory feedback mechanisms that limit excessive variations in shear stress responses. Additionally, endothelial cell-derived factors, such as nitric oxide and heparan sulfate proteoglycans, may counterbalance regional differences in flow to maintain a relatively uniform glycocalyx thickness (Moore et al., 2021). Based on shear stress equations [shear stress = fluid viscosity × fluid velocity/internal diameter] (Gnasso et al., 1996; Box et al., 2005), increasing lumen diameter can achieve a higher fluid velocity without increasing shear stress. If the AP diameter is larger in HF regions, as observed in SC in humans (Swain et al., 2022), then a higher flow rate could be achieved without increasing shear stress. This suggests that variations in aqueous outflow may be governed more by structural changes in outflow pathways than by glycocalyx-mediated resistance alone. This concept may extend to other outflow structures, such as CCs and ESVs. Future studies should investigate the size and total surface area of these structures in different flow regions and assess potential species-specific differences.

Apart from the coverage and thickness of the glycocalyx at different flow regions along the trabecular outflow pathway, the formation of the glycocalyx and its relationship with intraocular pressure should be further investigated. Yen et al. demonstrated that nitric oxide production in the cannulated rat mesentery increased when shear stress was elevated by increasing the perfusion flow velocity. This response was attenuated when the vessels were pretreated with an enzyme, heparanase III, to degrade glycocalyx (Yen et al., 2015). Similar findings have also been demonstrated in cultured aortic endothelial cells (Pahakis et al., 2007; Florian et al., 2003). These findings suggest that glycocalyx-mediated mechanosensing of shear stress may trigger downstream mechanotransduction pathways (e.g., nitric oxide production). Since nitric oxide enhances outflow facility and lowers intraocular pressure (Schneemann et al., 2002; Kotikoski et al., 2003; Dismuke et al., 2014; Muenster et al., 2017), glycocalyx-dependent mechanosensing may regulate aqueous outflow by initiating this downstream signaling response, which is crucial for maintaining intraocular pressure. Additionally, glycocalyx formation is restricted not only in the aged or diseased vessels (Targosz-Korecka et al., 2017; Salmon et al., 2012), but our previous study also showed that in ocular hypertensive eyes with 270° of TM damaged by photocoagulation burns (Sosnowik et al., 2022a), a minimal or absent glycocalyx was observed in the entire outflow pathway in the TM-damaged regions. These findings suggest a relationship among intraocular pressure regulation, outflow dynamics, and the synthesis of the glycocalyx. Glaucoma, which is the leading cause of irreversible blindness worldwide (GBD 2019 Blindness and Vision Impairment Collaborators, 2021), is associated with elevated intraocular pressure, a major risk factor for glaucoma (Weinreb et al., 2014). Lowering intraocular pressure is the primary clinical intervention for slowing the progression of glaucomatous visual field loss (The AGIS Investigators, 2000; Collaborative Normal-Tension Glaucoma Study Group, 1998; Leske et al., 2003). Increased outflow resistance in the trabecular outflow pathway is the primary cause of intraocular pressure elevation (Grant, 1963; Tamm and Fuchshofer, 2007). Hence, if the formation of the glycocalyx along the trabecular outflow pathway and consequently, nitric oxide is impaired, intraocular pressure regulation may be further disturbed in glaucoma patients.

In summary, our findings confirm that, similar to those in monkey and human eyes, glycocalyx thickness progressively increased from proximal to distal regions along the trabecular outflow pathway in bovine eyes. Glycocalyx was also observed in both I- and B-pores, with the thickest glycocalyx found at the pore centers. These results further support the relationship between aqueous humor flow and glycocalyx formation. However, while glycocalyx thickness did not differ significantly between HF and LF regions, it is possible that other factors also play an important role in modulating outflow resistance. Future studies should explore the interactions between glycocalyx formation and compositions, flow regions, and the morphologies of various outflow structures. These findings provide a morphological basis for future research on glycocalyx alterations in glaucoma and their impact on outflow resistance.

## Data Availability

The raw data supporting the conclusion of this article will be made available by the authors, without undue reservation.

## References

[B1] Arisaka T. Mitsumata M. Kawasumi M. Tohjima T. Hirose S. Yoshida Y. (1995). Effects of shear stress on glycosaminoglycan synthesis in vascular endothelial cells. Ann. N. Y. Acad. Sci. 748, 543–554. 10.1111/j.1749-6632.1994.tb17359.x 7695202

[B2] Bhatt K. Gong H. Freddo T. F. (1995). Freeze-fracture studies of interendothelial junctions in the angle of the human eye. Invest Ophthalmol. Vis. Sci. 36 (7), 1379–1389. 7775116

[B3] Bill A. (1966). Conventional and uveo-scleral drainage of aqueous humour in the cynomolgus monkey (Macaca irus) at normal and high intraocular pressures. Exp. Eye Res. 5 (1), 45–54. 10.1016/s0014-4835(66)80019-2 4160221

[B4] Bill A. Phillips C. I. (1971). Uveoscleral drainage of aqueous humour in human eyes. Exp. Eye Res. 12 (3), 275–281. 10.1016/0014-4835(71)90149-7 5130270

[B5] Box F. M. van der Geest R. J. Rutten M. C. Reiber J. H. (2005). The influence of flow, vessel diameter, and non-Newtonian blood viscosity on the wall shear stress in a carotid bifurcation model for unsteady flow. Invest Radiol. 40 (5), 277–294. 10.1097/01.rli.0000160550.95547.22 15829825

[B6] Braakman S. T. Read A. T. Chan D. W. Ethier C. R. Overby D. R. (2015). Colocalization of outflow segmentation and pores along the inner wall of Schlemm’s canal. Exp. Eye Res. 130, 87–96. 10.1016/j.exer.2014.11.008 25450060 PMC4305530

[B7] Cha E. D. K. Xu J. Gong L. Gong H. (2016). Variations in active outflow along the trabecular outflow pathway. Exp. Eye Res. 146, 354–360. 10.1016/j.exer.2016.01.008 26775054 PMC4893924

[B8] Chang J. Y. Folz S. J. Laryea S. N. Overby D. R. (2014). Multi-scale analysis of segmental outflow patterns in human trabecular meshwork with changing intraocular pressure. J. ocular Pharmacol. Ther. 30 (2-3), 213–223. 10.1089/jop.2013.0182 24456518

[B9] Chevalier L. Selim J. Castro C. Cuvilly F. Baste J. M. Richard V. (2022). Combined electron microscopy approaches for arterial glycocalyx visualization. Front. Cardiovasc Med. 9, 840689. 10.3389/fcvm.2022.840689 35355969 PMC8959549

[B10] Collaborative Normal-Tension Glaucoma Study Group (1998). Comparison of glaucomatous progression between untreated patients with normal-tension glaucoma and patients with therapeutically reduced intraocular pressures. Collaborative Normal-Tension Glaucoma Study Group. Am. J. Ophthalmol. 126 (4), 487–497. 10.1016/s0002-9394(98)00223-2 9780093

[B11] Cooper S. Emmott A. McDonald K. K. Campeau M. A. Leask R. L. (2018). Increased MMP activity in curved geometries disrupts the endothelial cell glycocalyx creating a proinflammatory environment. PLoS One 13 (8), e0202526. 10.1371/journal.pone.0202526 30138400 PMC6107195

[B12] Dismuke W. M. Liang J. Overby D. R. Stamer W. D. (2014). Concentration-related effects of nitric oxide and endothelin-1 on human trabecular meshwork cell contractility. Exp. Eye Res. 120, 28–35. 10.1016/j.exer.2013.12.012 24374036 PMC3943640

[B13] Ethier C. R. Coloma F. M. Sit A. J. Johnson M. (1998). Two pore types in the inner-wall endothelium of Schlemm’s canal. Invest Ophthalmol. Vis. Sci. 39 (11), 2041–2048. 9761282

[B14] Florian J. A. Kosky J. R. Ainslie K. Pang Z. Y. Dull R. O. Tarbell J. M. (2003). Heparan sulfate proteoglycan is a mechanosensor on endothelial cells. Circulation Res. 93 (10), E136–E142. 10.1161/01.RES.0000101744.47866.D5 14563712

[B15] Freddo T. F. Civan M. Gong H. (2022). “Aqueous humor and the dynamics of its flow: mechanisms and routes of aqueous humor drainage,” in Albert and Jakobiec’s Principles and Practice of Ophthalmology. Editors Albert D. M. Miller J. W. Azar D. T. Young L. H. (Cham: Springer International Publishing), 1989–2033.

[B16] Gao L. Lipowsky H. H. (2010). Composition of the endothelial glycocalyx and its relation to its thickness and diffusion of small solutes. Microvasc. Res. 80 (3), 394–401. 10.1016/j.mvr.2010.06.005 20600162 PMC2962421

[B17] GBD 2019 Blindness and Vision Impairment Collaborators Vision Loss Expert Group of the Global Burden of Disease Study (2021). Causes of blindness and vision impairment in 2020 and trends over 30 years, and prevalence of avoidable blindness in relation to VISION 2020: the Right to Sight: an analysis for the Global Burden of Disease Study. Lancet Glob. Health 9 (2), e144–e160. 10.1016/S2214-109X(20)30489-7 33275949 PMC7820391

[B18] Gnasso A. Carallo C. Irace C. Spagnuolo V. De Novara G. Mattioli P. L. (1996). Association between intima-media thickness and wall shear stress in common carotid arteries in healthy male subjects. Circulation 94 (12), 3257–3262. 10.1161/01.cir.94.12.3257 8989138

[B19] Gomez T. A. Golden G. J. Cummings R. D. Malmstrom J. Esko J. D. (2025). Endothelial glycocalyx turnover in vascular health and disease: rethinking endothelial dysfunction. Annu. Rev. Biochem. 10.1146/annurev-biochem-032620-104745 PMC13377631 40132227

[B20] Gong H. Francis A. (2014). Schlemm’s canal and collector channels as therapeutic targets. Surg. Innovations Glaucoma, 3–25. 10.1007/978-1-4614-8348-9_1

[B21] Gong H. Tripathi R. C. Tripathi B. J. (1996). Morphology of the aqueous outflow pathway. Microsc. Res. Tech. 33 (4), 336–367. 10.1002/(SICI)1097-0029(19960301)33:4<336::AID-JEMT4>3.0.CO;2-N 8652890

[B22] Grant W. M. (1963). Experimental aqueous perfusion in enucleated human eyes. Arch. Ophthalmol. 69, 783–801. 10.1001/archopht.1963.00960040789022 13949877

[B23] Haldenby K. A. Chappell D. C. Winlove C. P. Parker K. H. Firth J. A. (1994). Focal and regional variations in the composition of the glycocalyx of large vessel endothelium. J. Vasc. Res. 31 (1), 2–9. 10.1159/000159025 7506062

[B24] Huang A. S. Camp A. Xu B. Y. Penteado R. C. Weinreb R. N. (2017). Aqueous angiography: aqueous humor outflow imaging in live human subjects. Ophthalmology 124 (8), 1249–1251. 10.1016/j.ophtha.2017.03.058 28461013 PMC5522757

[B25] Huang A. S. Saraswathy S. Dastiridou A. Begian A. Legaspi H. Mohindroo C. (2016). Aqueous angiography with fluorescein and indocyanine green in bovine eyes. Transl. Vis. Sci. Technol. 5 (6), 5. 10.1167/tvst.5.6.5 PMC5106193 27847692

[B26] Johnson M. (2006). What controls aqueous humour outflow resistance? Exp. Eye Res. 82 (4), 545–557. 10.1016/j.exer.2005.10.011 16386733 PMC2892751

[B27] Kotikoski H. Vapaatalo H. Oksala O. (2003). Nitric oxide and cyclic GMP enhance aqueous humor outflow facility in rabbits. Curr. Eye Res. 26 (2), 119–123. 10.1076/ceyr.26.2.119.14511 12815531

[B28] Kutuzov N. Flyvbjerg H. Lauritzen M. (2018). Contributions of the glycocalyx, endothelium, and extravascular compartment to the blood-brain barrier. Proc. Natl. Acad. Sci. U. S. A. 115 (40), E9429–E9438. 10.1073/pnas.1802155115 30217895 PMC6176561

[B29] Lai J. Su Y. Swain D. L. Huang D. Getchevski D. Gong H. (2019). The role of schlemm’s canal endothelium cellular connectivity in giant vacuole formation: a 3D electron microscopy study. Invest Ophthalmol. Vis. Sci. 60 (5), 1630–1643. 10.1167/iovs.18-26011 30995299 PMC6736380

[B30] Leske M. C. Heijl A. Hussein M. Bengtsson B. Hyman L. Komaroff E. (2003). Factors for glaucoma progression and the effect of treatment: the early manifest glaucoma trial. Arch. Ophthalmol. 121 (1), 48–56. 10.1001/archopht.121.1.48 12523884

[B31] Mahmoud M. Mayer M. Cancel L. M. Bartosch A. M. Mathews R. Tarbell J. M. (2021). The glycocalyx core protein Glypican 1 protects vessel wall endothelial cells from stiffness-mediated dysfunction and disease. Cardiovasc Res. 117 (6), 1592–1605. 10.1093/cvr/cvaa201 32647868 PMC8152694

[B32] Moore K. H. Murphy H. A. George E. M. (2021). The glycocalyx: a central regulator of vascular function. Am. J. Physiol. Regul. Integr. Comp. Physiol. 320 (4), R508–R518. 10.1152/ajpregu.00340.2020 33501896 PMC8238147

[B33] Muenster S. Lieb W. S. Fabry G. Allen K. N. Kamat S. S. Guy A. H. (2017). The ability of nitric oxide to lower intraocular pressure is dependent on guanylyl cyclase. Investigative Ophthalmol. and Vis. Sci. 58 (11), 4826–4835. 10.1167/iovs.17-22168 PMC5624778 28973329

[B34] Pahakis M. Y. Kosky J. R. Dull R. O. Tarbell J. M. (2007). The role of endothelial glycocalyx components in mechanotransduction of fluid shear stress. Biochem. Bioph Res. Co. 355 (1), 228–233. 10.1016/j.bbrc.2007.01.137 PMC1847369 17291452

[B35] Raviola G. Raviola E. (1981). Paracellular route of aqueous outflow in the trabecular meshwork and canal of Schlemm. A freeze-fracture study of the endothelial junctions in the sclerocorneal angel of the macaque monkey eye. Invest Ophthalmol. Vis. Sci. 21 (1 Pt 1), 52–72. 7251302

[B36] Reitsma S. Slaaf D. W. Vink H. van Zandvoort M. A. oude Egbrink M. G. (2007). The endothelial glycocalyx: composition, functions, and visualization. Pflugers Arch. 454 (3), 345–359. 10.1007/s00424-007-0212-8 17256154 PMC1915585

[B37] Salmon A. H. Ferguson J. K. Burford J. L. Gevorgyan H. Nakano D. Harper S. J. (2012). Loss of the endothelial glycocalyx links albuminuria and vascular dysfunction. J. Am. Soc. Nephrol. 23 (8), 1339–1350. 10.1681/ASN.2012010017 22797190 PMC3402289

[B38] Schneemann A. Dijkstra B. G. van den Berg T. J. Kamphuis W. Hoyng P. F. (2002). Nitric oxide/guanylate cyclase pathways and flow in anterior segment perfusion. Graefes Arch. Clin. Exp. Ophthalmol. 240 (11), 936–941. 10.1007/s00417-002-0559-7 12486517

[B39] Sosnowik S. Swain D. L. Fan S. Toris C. B. Gong H. (2022b). Morphological changes to Schlemm’s canal and the distal aqueous outflow pathway in monkey eyes with laser-induced ocular hypertension. Exp. Eye Res. 219, 109030. 10.1016/j.exer.2022.109030 35283108 PMC9133064

[B40] Sosnowik S. Swain D. L. Liu N. Fan S. Toris C. B. Gong H. (2022a). Endothelial glycocalyx morphology in different flow regions of the aqueous outflow pathway of normal and laser-induced glaucoma monkey eyes. Cells 11 (15), 2452. 10.3390/cells11152452 35954296 PMC9367875

[B41] Swain D. L. Le T. D. Yasmin S. Fernandes B. Lamaj G. Dasgupta I. (2021). Morphological factors associated with giant vacuoles with I-pores in Schlemm’s canal endothelial cells of human eyes: a serial block-face scanning electron microscopy study. Exp. Eye Res. 205, 108488. 10.1016/j.exer.2021.108488 33571532 PMC8044056

[B42] Swain D. L. Yasmin S. Fernandes B. Lamaj G. Su Y. Gong H. (2022). Schlemm’s canal endothelium cellular connectivity in giant vacuole and pore formation in different flow-type areas: a serial block-face scanning electron microscopy study. Front. Cell Dev. Biol. 10, 867376. 10.3389/fcell.2022.867376 35493087 PMC9043561

[B43] Tamm E. R. Fuchshofer R. (2007). What increases outflow resistance in primary open-angle glaucoma? Surv. Ophthalmol. 52, S101–S104. 10.1016/j.survophthal.2007.08.002 17998032

[B44] Targosz-Korecka M. Jaglarz M. Malek-Zietek K. E. Gregorius A. Zakrzewska A. Sitek B. (2017). AFM-based detection of glycocalyx degradation and endothelial stiffening in the db/db mouse model of diabetes. Sci. Rep. 7 (1), 15951. 10.1038/s41598-017-16179-7 29162916 PMC5698475

[B45] The AGIS Investigators (2000). The Advanced Glaucoma Intervention Study (AGIS): 7. The relationship between control of intraocular pressure and visual field deterioration.The AGIS Investigators. Am. J. Ophthalmol. 130 (4), 429–440. 10.1016/s0002-9394(00)00538-9 11024415

[B46] Tripathi R. C. (1971a). Ultrastructure of the exit pathway of the aqueous in lower mammals. (A preliminary report on the “angular aqueous plexus”). Exp. Eye Res. 12 (3), 311–314. 10.1016/0014-4835(71)90155-2 5130275

[B47] Tripathi R. C. (1971b). Mechanism of the aqueous outflow across the trabecular wall of Schlemm’s canal. Exp. Eye Res. 11 (1), 116–121. 10.1016/s0014-4835(71)80073-8 4108660

[B48] Tripathi R. C. (1977). The functional morphology of the outflow systems of ocular and cerebrospinal fluids. Exp. Eye Res. 25 (Suppl. l), 65–116. 10.1016/s0014-4835(77)80010-9 590403

[B49] van den Berg B. M. Vink H. Spaan J. A. (2003). The endothelial glycocalyx protects against myocardial edema. Circ. Res. 92 (6), 592–594. 10.1161/01.RES.0000065917.53950.75 12637366

[B50] van Haaren P. M. VanBavel E. Vink H. Spaan J. A. (2003). Localization of the permeability barrier to solutes in isolated arteries by confocal microscopy. Am. J. Physiol. Heart Circ. Physiol. 285 (6), H2848–H2856. 10.1152/ajpheart.00117.2003 12907418

[B51] Vranka J. A. Bradley J. M. Yang Y. F. Keller K. E. Acott T. S. (2015). Mapping molecular differences and extracellular matrix gene expression in segmental outflow pathways of the human ocular trabecular meshwork. PLoS One 10 (3), e0122483. 10.1371/journal.pone.0122483 25826404 PMC4380331

[B52] Wang G. Kostidis S. Tiemeier G. L. Sol W. de Vries M. R. Giera M. (2020). Shear stress regulation of endothelial glycocalyx structure is determined by glucobiosynthesis. Arterioscler. Thromb. Vasc. Biol. 40 (2), 350–364. 10.1161/ATVBAHA.119.313399 31826652

[B53] Weinbaum S. Tarbell J. M. Damiano E. R. (2007). The structure and function of the endothelial glycocalyx layer. Annu. Rev. Biomed. Eng. 9, 121–167. 10.1146/annurev.bioeng.9.060906.151959 17373886

[B54] Weinbaum S. Zhang X. Han Y. Vink H. Cowin S. C. (2003). Mechanotransduction and flow across the endothelial glycocalyx. Proc. Natl. Acad. Sci. U. S. A. 100 (13), 7988–7995. 10.1073/pnas.1332808100 12810946 PMC164700

[B55] Weinreb R. N. Aung T. Medeiros F. A. (2014). The pathophysiology and treatment of glaucoma: a review. JAMA 311 (18), 1901–1911. 10.1001/jama.2014.3192 24825645 PMC4523637

[B56] Yang C. Y. Huynh T. Johnson M. Gong H. (2014). Endothelial glycocalyx layer in the aqueous outflow pathway of bovine and human eyes. Exp. Eye Res. 128, 27–33. 10.1016/j.exer.2014.08.015 25217864 PMC4254369

[B57] Yang Y. Haeger S. M. Suflita M. A. Zhang F. Dailey K. L. Colbert J. F. (2017). Fibroblast growth factor signaling mediates pulmonary endothelial glycocalyx reconstitution. Am. J. Respir. Cell Mol. Biol. 56 (6), 727–737. 10.1165/rcmb.2016-0338OC 28187268 PMC5516293

[B58] Yen W. Y. Cai B. Yang J. L. Zhang L. Zeng M. Tarbell J. M. (2015). Endothelial surface glycocalyx can regulate flow-induced nitric oxide production in microvessels *in vivo* . PLoS One 10 (1), e0117133. 10.1371/journal.pone.0117133 25575016 PMC4289188

[B59] Yen W. Y. Cai B. Zeng M. Tarbell J. M. Fu B. M. (2012). Quantification of the endothelial surface glycocalyx on rat and mouse blood vessels. Microvasc. Res. 83 (3), 337–346. 10.1016/j.mvr.2012.02.005 22349291 PMC3371771

[B60] Zeng Y. Tarbell J. M. (2014). The adaptive remodeling of endothelial glycocalyx in response to fluid shear stress. PLoS One 9 (1), e86249. 10.1371/journal.pone.0086249 24465988 PMC3896483

